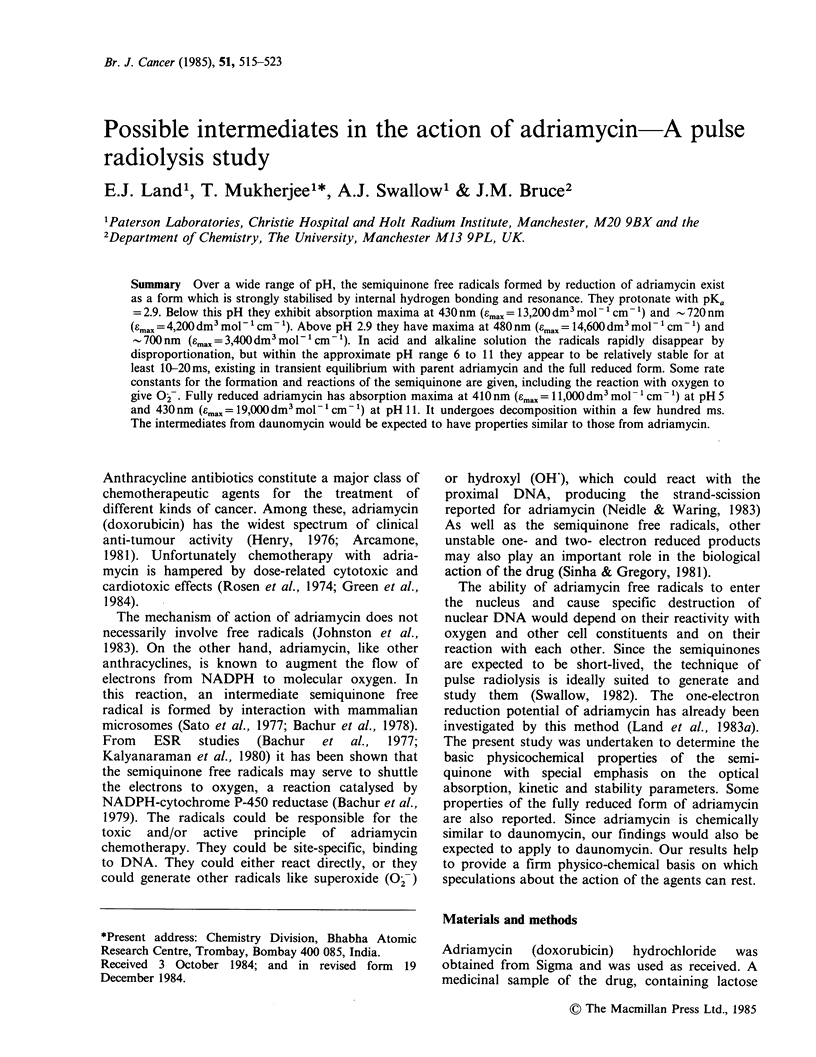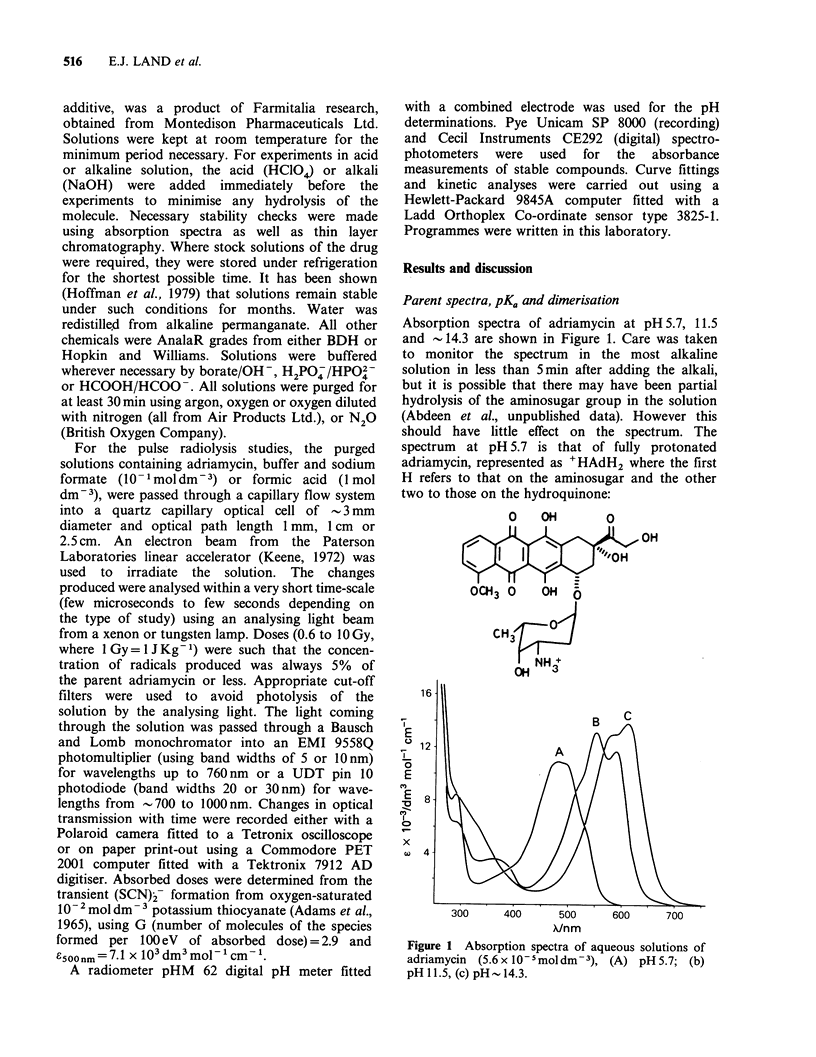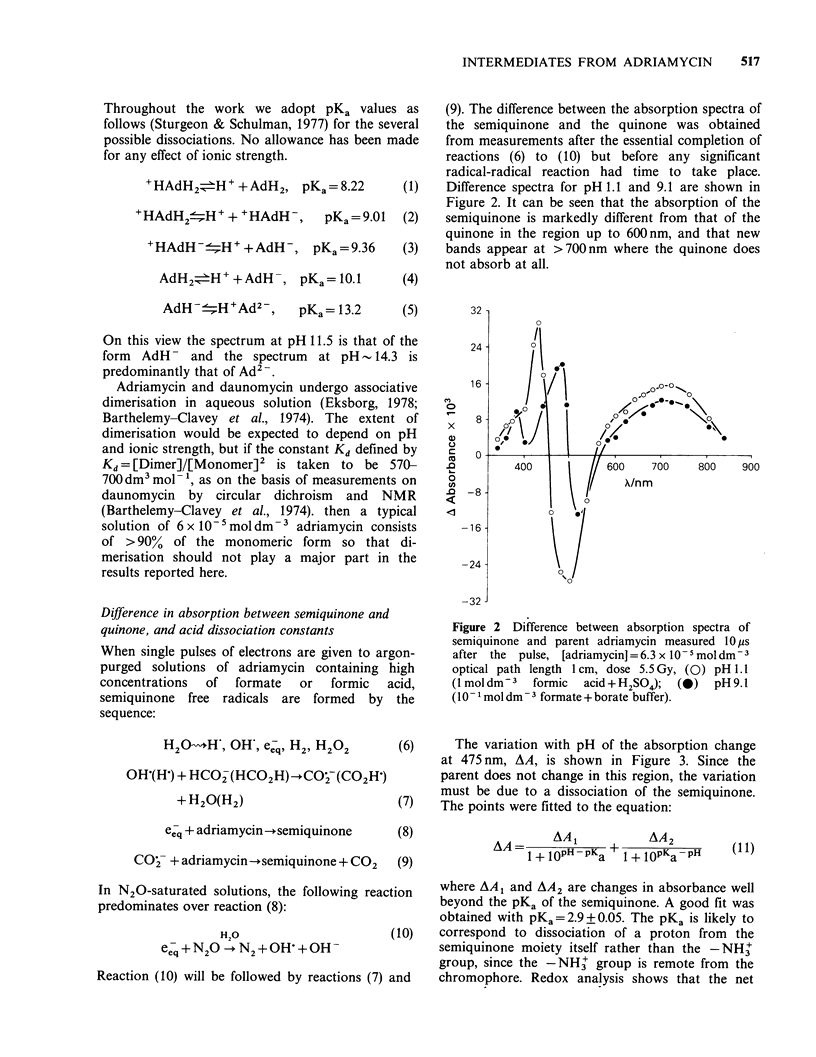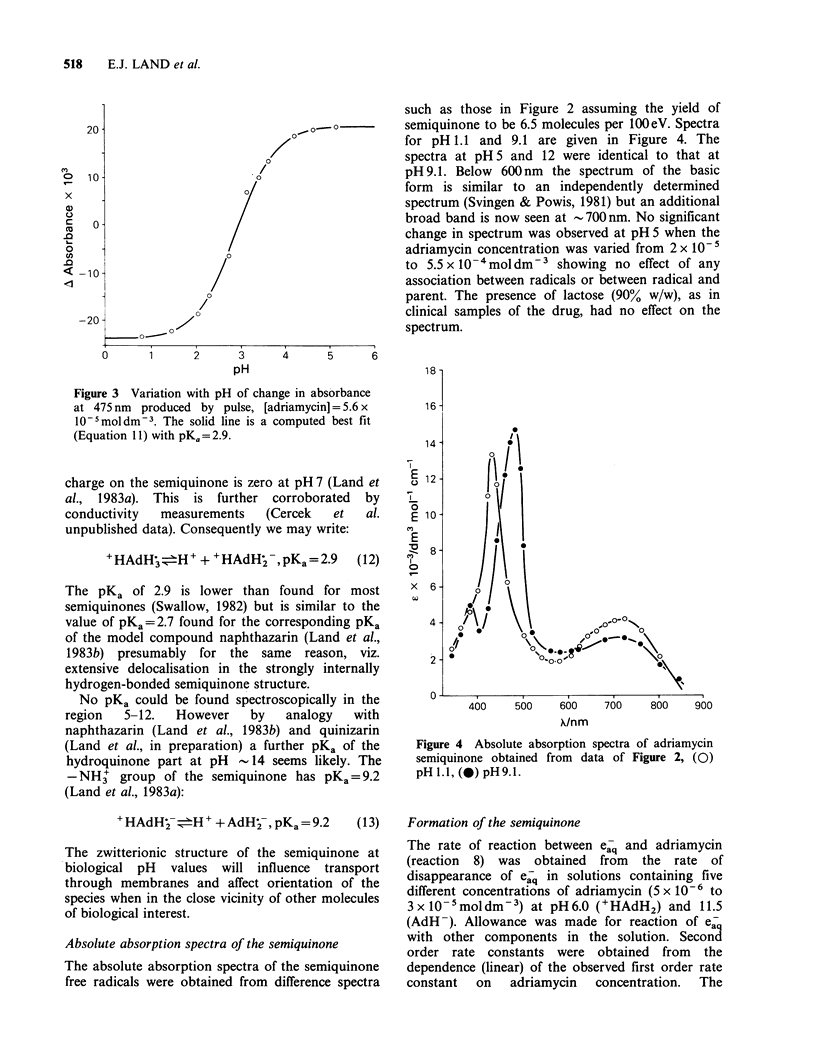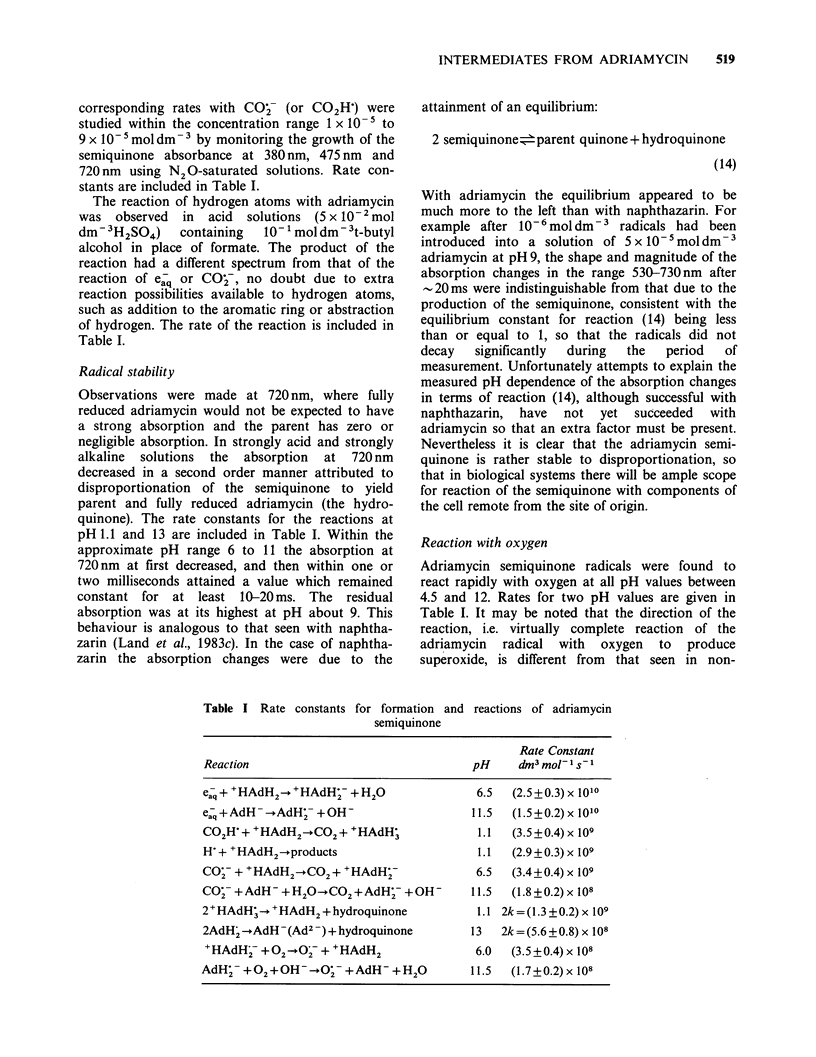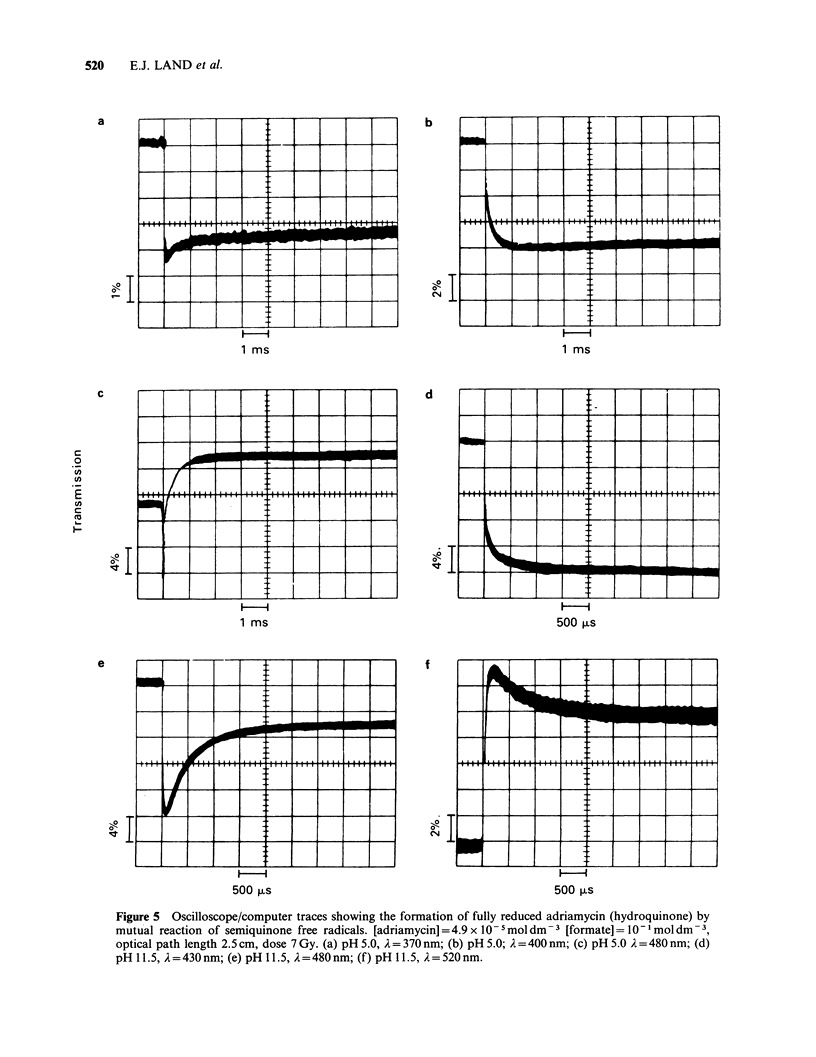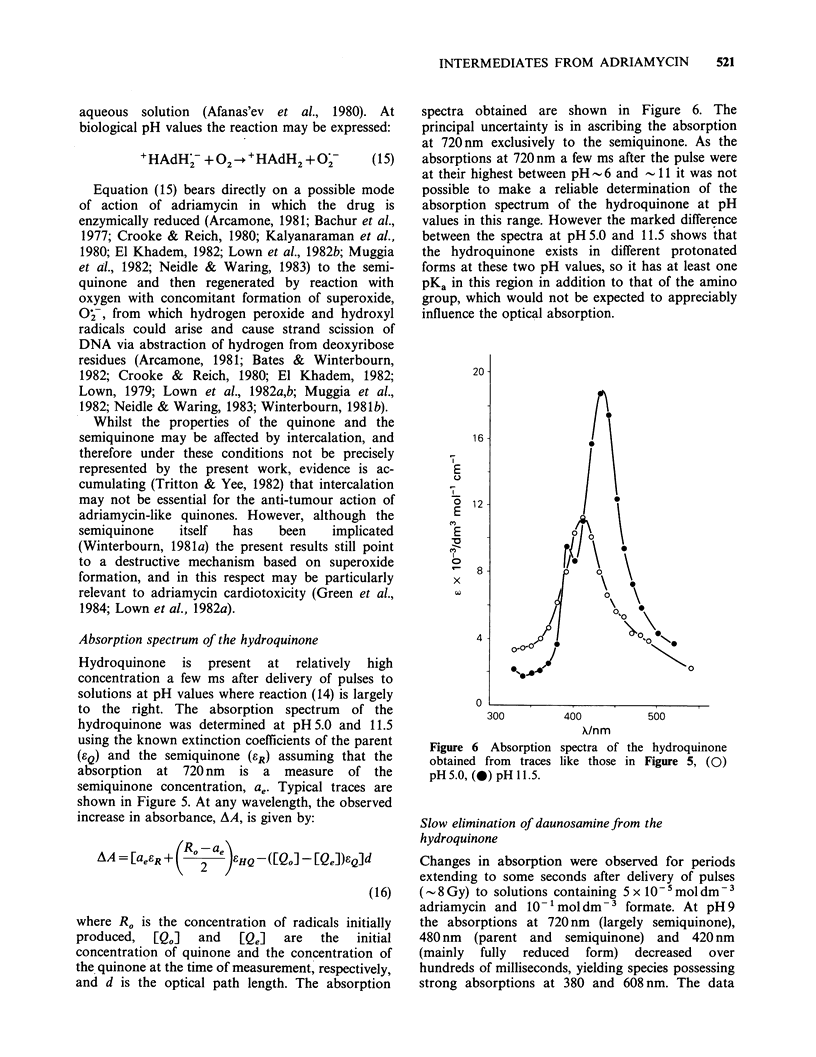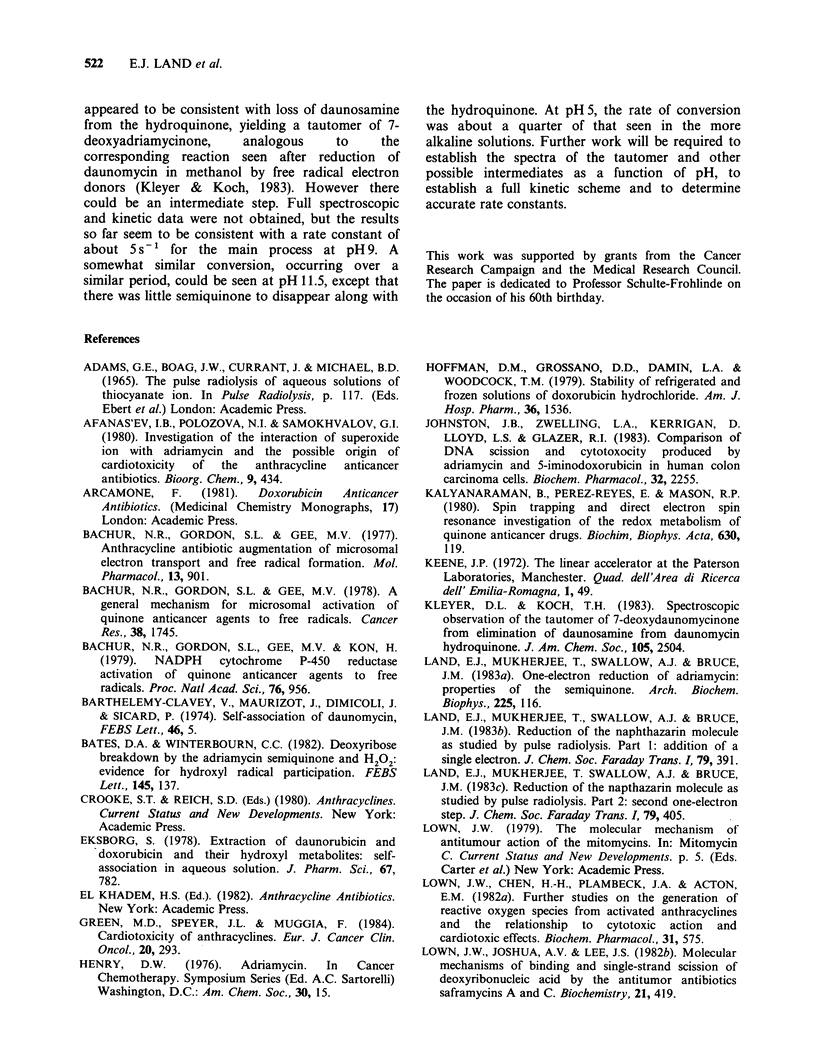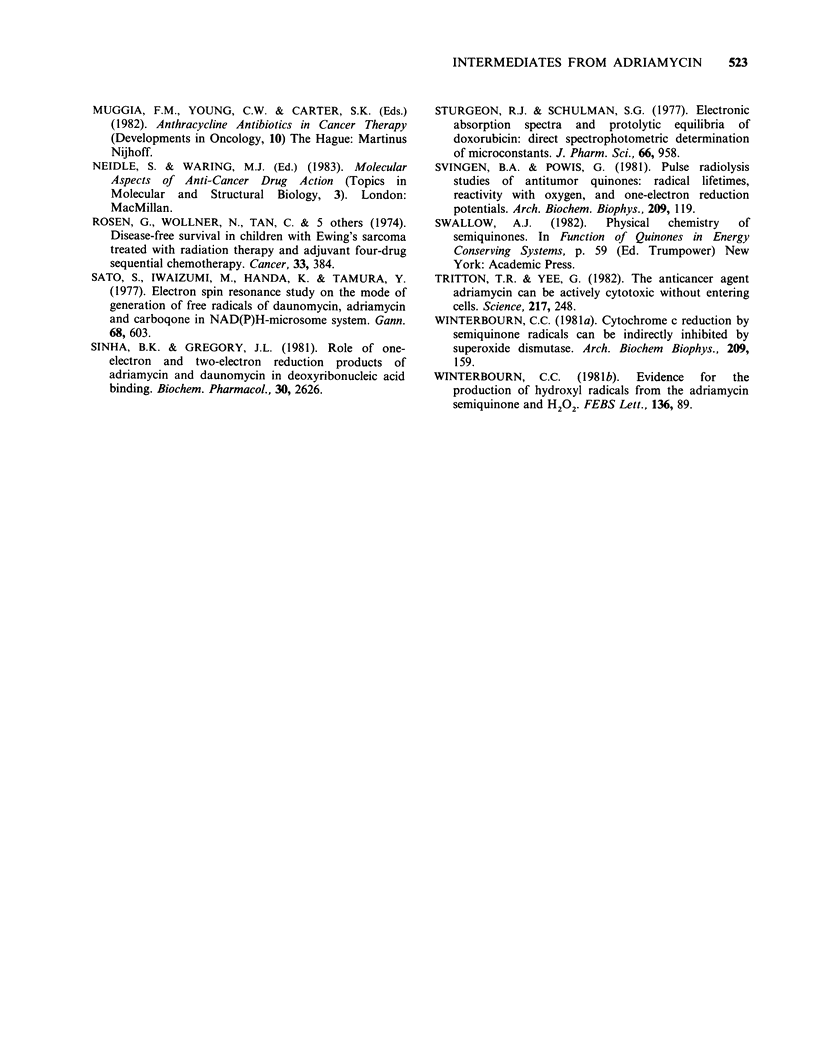# Possible intermediates in the action of adriamycin--a pulse radiolysis study.

**DOI:** 10.1038/bjc.1985.74

**Published:** 1985-04

**Authors:** E. J. Land, T. Mukherjee, A. J. Swallow, J. M. Bruce

## Abstract

Over a wide range of pH, the semiquinone free radicals formed by reduction of adriamycin exist as a form which is strongly stabilised by internal hydrogen bonding and resonance. They protonate with pKa = 2.9. Below this pH they exhibit absorption maxima at 430 nm (Emax = 13,200 dm3 mol-1 cm-1) and approximately 720 nm (Emax = 4,200 dm3 mol-1 cm-1). Above pH 2.9 they have maxima at 480 nm (Emax = 14,600 dm3 mol-1 cm-1) and approximately 700 nm (Emax = 3,400 dm3 mol-1 cm-1). In acid and alkaline solution the radicals rapidly disappear by disproportionation, but within the approximate pH range 6 to 11 they appear to be relatively stable for at least 10-20 ms, existing in transient equilibrium with parent adriamycin and the full reduced form. Some rate constants for the formation and reactions of the semiquinone are given, including the reaction with oxygen to give O2.-. Fully reduced adriamycin has absorption maxima at 410 nm (Emax = 11,000 dm3 mol-1 cm-1) at pH 5 and 430 nm (Emax = 19,000 dm3 mol-1 cm-1) at pH 11. It undergoes decomposition within a few hundred ms. The intermediates from daunomycin would be expected to have properties similar to those from adriamycin.


*Br. J. Cancer* (1985), **51**, 515–523

### Possible intermediates in the action of adriamycin—A pulse radiolysis study

E.J. Land[1], T. Mukherjee[1]*, A.J. Swallow[1] & J.M. Bruce[2]

[1]*Paterson Laboratories, Christie Hospital and Holt Radium Institute, Manchester, M20 9BX and the* [2]*Department of Chemistry, The University, Manchester M13 9PL, UK.*

**Summary** Over a wide range of pH, the semiquinone free radicals formed by reduction of adriamycin exist as a form which is strongly stabilised by internal hydrogen bonding and resonance. They protonate with $pK_a = 2.9$. Below this pH they exhibit absorption maxima at 430 nm ($\varepsilon_{max} = 13{,}200\,dm^3\,mol^{-1}\,cm^{-1}$) and ~720 nm ($\varepsilon_{max} = 4{,}200\,dm^3\,mol^{-1}\,cm^{-1}$). Above pH 2.9 they have maxima at 480 nm ($\varepsilon_{max} = 14{,}600\,dm^3\,mol^{-1}\,cm^{-1}$) and ~700 nm ($\varepsilon_{max} = 3{,}400\,dm^3\,mol^{-1}\,cm^{-1}$). In acid and alkaline solution the radicals rapidly disappear by disproportionation, but within the approximate pH range 6 to 11 they appear to be relatively stable for at least 10–20 ms, existing in transient equilibrium with parent adriamycin and the full reduced form. Some rate constants for the formation and reactions of the semiquinone are given, including the reaction with oxygen to give $O_2^{\cdot-}$. Fully reduced adriamycin has absorption maxima at 410 nm ($\varepsilon_{max} = 11{,}000\,dm^3\,mol^{-1}\,cm^{-1}$) at pH 5 and 430 nm ($\varepsilon_{max} = 19{,}000\,dm^3\,mol^{-1}\,cm^{-1}$) at pH 11. It undergoes decomposition within a few hundred ms. The intermediates from daunomycin would be expected to have properties similar to those from adriamycin.

Anthracycline antibiotics constitute a major class of chemotherapeutic agents for the treatment of different kinds of cancer. Among these, adriamycin (doxorubicin) has the widest spectrum of clinical anti-tumour activity (Henry, 1976; Arcamone, 1981). Unfortunately chemotherapy with adriamycin is hampered by dose-related cytotoxic and cardiotoxic effects (Rosen *et al.*, 1974; Green *et al.*, 1984).

The mechanism of action of adriamycin does not necessarily involve free radicals (Johnston *et al.*, 1983). On the other hand, adriamycin, like other anthracyclines, is known to augment the flow of electrons from NADPH to molecular oxygen. In this reaction, an intermediate semiquinone free radical is formed by interaction with mammalian microsomes (Sato *et al.*, 1977; Bachur *et al.*, 1978). From ESR studies (Bachur *et al.*, 1977; Kalyanaraman *et al.*, 1980) it has been shown that the semiquinone free radicals may serve to shuttle the electrons to oxygen, a reaction catalysed by NADPH-cytochrome P-450 reductase (Bachur *et al.*, 1979). The radicals could be responsible for the toxic and/or active principle of adriamycin chemotherapy. They could be site-specific, binding to DNA. They could either react directly, or they could generate other radicals like superoxide ($O_2^{\cdot-}$) or hydroxyl ($OH^{\cdot}$), which could react with the proximal DNA, producing the strand-scission reported for adriamycin (Neidle & Waring, 1983) As well as the semiquinone free radicals, other unstable one- and two- electron reduced products may also play an important role in the biological action of the drug (Sinha & Gregory, 1981).

The ability of adriamycin free radicals to enter the nucleus and cause specific destruction of nuclear DNA would depend on their reactivity with oxygen and other cell constituents and on their reaction with each other. Since the semiquinones are expected to be short-lived, the technique of pulse radiolysis is ideally suited to generate and study them (Swallow, 1982). The one-electron reduction potential of adriamycin has already been investigated by this method (Land *et al.*, 1983*a*). The present study was undertaken to determine the basic physicochemical properties of the semiquinone with special emphasis on the optical absorption, kinetic and stability parameters. Some properties of the fully reduced form of adriamycin are also reported. Since adriamycin is chemically similar to daunomycin, our findings would also be expected to apply to daunomycin. Our results help to provide a firm physico-chemical basis on which speculations about the action of the agents can rest.

### Materials and methods

Adriamycin (doxorubicin) hydrochloride was obtained from Sigma and was used as received. A medicinal sample of the drug, containing lactose

*Present address: Chemistry Division, Bhabha Atomic Research Centre, Trombay, Bombay 400 085, India.
Received 3 October 1984; and in revised form 19 December 1984.

© The Macmillan Press Ltd., 1985

additive, was a product of Farmitalia research, obtained from Montedison Pharmaceuticals Ltd. Solutions were kept at room temperature for the minimum period necessary. For experiments in acid or alkaline solution, the acid ($HClO_4$) or alkali (NaOH) were added immediately before the experiments to minimise any hydrolysis of the molecule. Necessary stability checks were made using absorption spectra as well as thin layer chromatography. Where stock solutions of the drug were required, they were stored under refrigeration for the shortest possible time. It has been shown (Hoffman *et al.*, 1979) that solutions remain stable under such conditions for months. Water was redistilled from alkaline permanganate. All other chemicals were AnalaR grades from either BDH or Hopkin and Williams. Solutions were buffered wherever necessary by borate/$OH^-$, $H_2PO_4^-/HPO_4^{2-}$ or $HCOOH/HCOO^-$. All solutions were purged for at least 30 min using argon, oxygen or oxygen diluted with nitrogen (all from Air Products Ltd.), or $N_2O$ (British Oxygen Company).

For the pulse radiolysis studies, the purged solutions containing adriamycin, buffer and sodium formate ($10^{-1}$ mol dm$^{-3}$) or formic acid (1 mol dm$^{-3}$), were passed through a capillary flow system into a quartz capillary optical cell of ~3 mm diameter and optical path length 1 mm, 1 cm or 2.5 cm. An electron beam from the Paterson Laboratories linear accelerator (Keene, 1972) was used to irradiate the solution. The changes produced were analysed within a very short time-scale (few microseconds to few seconds depending on the type of study) using an analysing light beam from a xenon or tungsten lamp. Doses (0.6 to 10 Gy, where 1 Gy = 1 J Kg$^{-1}$) were such that the concentration of radicals produced was always 5% of the parent adriamycin or less. Appropriate cut-off filters were used to avoid photolysis of the solution by the analysing light. The light coming through the solution was passed through a Bausch and Lomb monochromator into an EMI 9558Q photomultiplier (using band widths of 5 or 10 nm) for wavelengths up to 760 nm or a UDT pin 10 photodiode (band widths 20 or 30 nm) for wavelengths from ~700 to 1000 nm. Changes in optical transmission with time were recorded either with a Polaroid camera fitted to a Tetronix oscilloscope or on paper print-out using a Commodore PET 2001 computer fitted with a Tektronix 7912 AD digitiser. Absorbed doses were determined from the transient $(SCN)_2^{-}$ formation from oxygen-saturated $10^{-2}$ mol dm$^{-3}$ potassium thiocyanate (Adams *et al.*, 1965), using G (number of molecules of the species formed per 100 eV of absorbed dose) = 2.9 and $\varepsilon_{500nm} = 7.1 \times 10^3$ dm$^3$ mol$^{-1}$ cm$^{-1}$.

A radiometer pHM 62 digital pH meter fitted with a combined electrode was used for the pH determinations. Pye Unicam SP 8000 (recording) and Cecil Instruments CE292 (digital) spectrophotometers were used for the absorbance measurements of stable compounds. Curve fittings and kinetic analyses were carried out using a Hewlett-Packard 9845A computer fitted with a Ladd Orthoplex Co-ordinate sensor type 3825-1. Programmes were written in this laboratory.

### Results and discussion

### *Parent spectra, $pK_a$ and dimerisation*

Absorption spectra of adriamycin at pH 5.7, 11.5 and ~14.3 are shown in Figure 1. Care was taken to monitor the spectrum in the most alkaline solution in less than 5 min after adding the alkali, but it is possible that there may have been partial hydrolysis of the aminosugar group in the solution (Abdeen *et al.*, unpublished data). However this should have little effect on the spectrum. The spectrum at pH 5.7 is that of fully protonated adriamycin, represented as $^+HAdH_2$ where the first H refers to that on the aminosugar and the other two to those on the hydroquinone:

**Figure 1** Absorption spectra of aqueous solutions of adriamycin ($5.6 \times 10^{-5}$ mol dm$^{-3}$), (A) pH 5.7; (b) pH 11.5, (c) pH ~ 14.3.

Throughout the work we adopt $pK_a$ values as follows (Sturgeon & Schulman, 1977) for the several possible dissociations. No allowance has been made for any effect of ionic strength.

$$^{+}HAdH_2 \rightleftharpoons H^+ + AdH_2, \quad pK_a = 8.22 \qquad (1)$$

$$^{+}HAdH_2 \rightleftharpoons H^+ + {}^{+}HAdH^-, \quad pK_a = 9.01 \qquad (2)$$

$$^{+}HAdH^- \rightleftharpoons H^+ + AdH^-, \quad pK_a = 9.36 \qquad (3)$$

$$AdH_2 \rightleftharpoons H^+ + AdH^-, \quad pK_a = 10.1 \qquad (4)$$

$$AdH^- \rightleftharpoons H^+ Ad^{2-}, \quad pK_a = 13.2 \qquad (5)$$

On this view the spectrum at pH 11.5 is that of the form $AdH^-$ and the spectrum at pH ~ 14.3 is predominantly that of $Ad^{2-}$.

Adriamycin and daunomycin undergo associative dimerisation in aqueous solution (Eksborg, 1978; Barthelemy–Clavey *et al.*, 1974). The extent of dimerisation would be expected to depend on pH and ionic strength, but if the constant $K_d$ defined by $K_d = [\text{Dimer}]/[\text{Monomer}]^2$ is taken to be 570–700 $dm^3 mol^{-1}$, as on the basis of measurements on daunomycin by circular dichroism and NMR (Barthelemy–Clavey *et al.*, 1974). then a typical solution of $6 \times 10^{-5} mol\,dm^{-3}$ adriamycin consists of >90% of the monomeric form so that dimerisation should not play a major part in the results reported here.

*Difference in absorption between semiquinone and quinone, and acid dissociation constants*

When single pulses of electrons are given to argon-purged solutions of adriamycin containing high concentrations of formate or formic acid, semiquinone free radicals are formed by the sequence:

$$H_2O \rightsquigarrow H^{\cdot}, OH^{\cdot}, e^-_{eq}, H_2, H_2O_2 \qquad (6)$$

$$OH^{\cdot}(H^{\cdot}) + HCO_2^-(HCO_2H) \rightarrow CO_2^{\cdot-}(CO_2H^{\cdot}) + H_2O(H_2) \qquad (7)$$

$$e^-_{eq} + \text{adriamycin} \rightarrow \text{semiquinone} \qquad (8)$$

$$CO_2^{\cdot-} + \text{adriamycin} \rightarrow \text{semiquinone} + CO_2 \qquad (9)$$

In $N_2O$-saturated solutions, the following reaction predominates over reaction (8):

$$e^-_{eq} + N_2O \xrightarrow{H_2O} N_2 + OH^{\cdot} + OH^- \qquad (10)$$

Reaction (10) will be followed by reactions (7) and (9). The difference between the absorption spectra of the semiquinone and the quinone was obtained from measurements after the essential completion of reactions (6) to (10) but before any significant radical-radical reaction had time to take place. Difference spectra for pH 1.1 and 9.1 are shown in Figure 2. It can be seen that the absorption of the semiquinone is markedly different from that of the quinone in the region up to 600 nm, and that new bands appear at >700 nm where the quinone does not absorb at all.

**Figure 2** Difference between absorption spectra of semiquinone and parent adriamycin measured 10 μs after the pulse, [adriamycin] = $6.3 \times 10^{-5} mol\,dm^{-3}$ optical path length 1 cm, dose 5.5 Gy, (○) pH 1.1 (1 $mol\,dm^{-3}$ formic acid + $H_2SO_4$); (●) pH 9.1 ($10^{-1} mol\,dm^{-3}$ formate + borate buffer).

The variation with pH of the absorption change at 475 nm, $\Delta A$, is shown in Figure 3. Since the parent does not change in this region, the variation must be due to a dissociation of the semiquinone. The points were fitted to the equation:

$$\Delta A = \frac{\Delta A_1}{1 + 10^{pH - pK_a}} + \frac{\Delta A_2}{1 + 10^{pK_a - pH}} \qquad (11)$$

where $\Delta A_1$ and $\Delta A_2$ are changes in absorbance well beyond the $pK_a$ of the semiquinone. A good fit was obtained with $pK_a = 2.9 \pm 0.05$. The $pK_a$ is likely to correspond to dissociation of a proton from the semiquinone moiety itself rather than the $-NH_3^+$ group, since the $-NH_3^+$ group is remote from the chromophore. Redox analysis shows that the net

**Figure 3** Variation with pH of change in absorbance at 475 nm produced by pulse, [adriamycin] $= 5.6 \times 10^{-5}$ mol dm$^{-3}$. The solid line is a computed best fit (Equation 11) with $pK_a = 2.9$.

charge on the semiquinone is zero at pH 7 (Land *et al.*, 1983*a*). This is further corroborated by conductivity measurements (Cercek *et al.* unpublished data). Consequently we may write:

$$^{+}HAdH_3^{\cdot} \rightleftharpoons H^{+} + {}^{+}HAdH_2^{\cdot -}, pK_a = 2.9 \quad (12)$$

The $pK_a$ of 2.9 is lower than found for most semiquinones (Swallow, 1982) but is similar to the value of $pK_a = 2.7$ found for the corresponding $pK_a$ of the model compound naphthazarin (Land *et al.*, 1983*b*) presumably for the same reason, viz. extensive delocalisation in the strongly internally hydrogen-bonded semiquinone structure.

No $pK_a$ could be found spectroscopically in the region 5–12. However by analogy with naphthazarin (Land *et al.*, 1983*b*) and quinizarin (Land *et al.*, in preparation) a further $pK_a$ of the hydroquinone part at pH $\sim$14 seems likely. The $-NH_3^+$ group of the semiquinone has $pK_a = 9.2$ (Land *et al.*, 1983*a*):

$$^{+}HAdH_2^{\cdot -} \rightleftharpoons H^{+} + AdH_2^{\cdot -}, pK_a = 9.2 \quad (13)$$

The zwitterionic structure of the semiquinone at biological pH values will influence transport through membranes and affect orientation of the species when in the close vicinity of other molecules of biological interest.

*Absolute absorption spectra of the semiquinone*

The absolute absorption spectra of the semiquinone free radicals were obtained from difference spectra such as those in Figure 2 assuming the yield of semiquinone to be 6.5 molecules per 100 eV. Spectra for pH 1.1 and 9.1 are given in Figure 4. The spectra at pH 5 and 12 were identical to that at pH 9.1. Below 600 nm the spectrum of the basic form is similar to an independently determined spectrum (Svingen & Powis, 1981) but an additional broad band is now seen at $\sim$700 nm. No significant change in spectrum was observed at pH 5 when the adriamycin concentration was varied from $2 \times 10^{-5}$ to $5.5 \times 10^{-4}$ mol dm$^{-3}$ showing no effect of any association between radicals or between radical and parent. The presence of lactose (90% w/w), as in clinical samples of the drug, had no effect on the spectrum.

**Figure 4** Absolute absorption spectra of adriamycin semiquinone obtained from data of **Figure 2**, (○) pH 1.1, (●) pH 9.1.

*Formation of the semiquinone*

The rate of reaction between $e_{aq}^-$ and adriamycin (reaction 8) was obtained from the rate of disappearance of $e_{aq}^-$ in solutions containing five different concentrations of adriamycin ($5 \times 10^{-6}$ to $3 \times 10^{-5}$ mol dm$^{-3}$) at pH 6.0 ($^{+}HAdH_2$) and 11.5 ($AdH^-$). Allowance was made for reaction of $e_{aq}^-$ with other components in the solution. Second order rate constants were obtained from the dependence (linear) of the observed first order rate constant on adriamycin concentration. The corresponding rates with $CO_2^{\cdot-}$ (or $CO_2H^{\cdot}$) were studied within the concentration range $1 \times 10^{-5}$ to $9 \times 10^{-5}$ mol dm$^{-3}$ by monitoring the growth of the semiquinone absorbance at 380 nm, 475 nm and 720 nm using $N_2O$-saturated solutions. Rate constants are included in Table I.

The reaction of hydrogen atoms with adriamycin was observed in acid solutions ($5 \times 10^{-2}$ mol dm$^{-3}$ $H_2SO_4$) containing $10^{-1}$ mol dm$^{-3}$ t-butyl alcohol in place of formate. The product of the reaction had a different spectrum from that of the reaction of $e_{aq}^-$ or $CO_2^{\cdot-}$, no doubt due to extra reaction possibilities available to hydrogen atoms, such as addition to the aromatic ring or abstraction of hydrogen. The rate of the reaction is included in Table I.

*Radical stability*

Observations were made at 720 nm, where fully reduced adriamycin would not be expected to have a strong absorption and the parent has zero or negligible absorption. In strongly acid and strongly alkaline solutions the absorption at 720 nm decreased in a second order manner attributed to disproportionation of the semiquinone to yield parent and fully reduced adriamycin (the hydroquinone). The rate constants for the reactions at pH 1.1 and 13 are included in Table I. Within the approximate pH range 6 to 11 the absorption at 720 nm at first decreased, and then within one or two milliseconds attained a value which remained constant for at least 10–20 ms. The residual absorption was at its highest at pH about 9. This behaviour is analogous to that seen with naphthazarin (Land *et al.*, 1983*c*). In the case of naphthazarin the absorption changes were due to the attainment of an equilibrium:

$$2 \text{ semiquinone} \rightleftharpoons \text{parent quinone} + \text{hydroquinone} \quad (14)$$

With adriamycin the equilibrium appeared to be much more to the left than with naphthazarin. For example after $10^{-6}$ mol dm$^{-3}$ radicals had been introduced into a solution of $5 \times 10^{-5}$ mol dm$^{-3}$ adriamycin at pH 9, the shape and magnitude of the absorption changes in the range 530–730 nm after ~20 ms were indistinguishable from that due to the production of the semiquinone, consistent with the equilibrium constant for reaction (14) being less than or equal to 1, so that the radicals did not decay significantly during the period of measurement. Unfortunately attempts to explain the measured pH dependence of the absorption changes in terms of reaction (14), although successful with naphthazarin, have not yet succeeded with adriamycin so that an extra factor must be present. Nevertheless it is clear that the adriamycin semiquinone is rather stable to disproportionation, so that in biological systems there will be ample scope for reaction of the semiquinone with components of the cell remote from the site of origin.

*Reaction with oxygen*

Adriamycin semiquinone radicals were found to react rapidly with oxygen at all pH values between 4.5 and 12. Rates for two pH values are given in Table I. It may be noted that the direction of the reaction, i.e. virtually complete reaction of the adriamycin radical with oxygen to produce superoxide, is different from that seen in non-

**Table I** Rate constants for formation and reactions of adriamycin semiquinone

| *Reaction* | *pH* | *Rate Constant* $dm^3\,mol^{-1}\,s^{-1}$ |
|---|---|---|
| $e_{aq}^- + {}^+HAdH_2 \rightarrow {}^+HAdH_2^{\cdot-} + H_2O$ | 6.5 | $(2.5 \pm 0.3) \times 10^{10}$ |
| $e_{aq}^- + AdH^- \rightarrow AdH_2^{\cdot-} + OH^-$ | 11.5 | $(1.5 \pm 0.2) \times 10^{10}$ |
| $CO_2H^{\cdot} + {}^+HAdH_2 \rightarrow CO_2 + {}^+HAdH_3^{\cdot}$ | 1.1 | $(3.5 \pm 0.4) \times 10^9$ |
| $H^{\cdot} + {}^+HAdH_2 \rightarrow$ products | 1.1 | $(2.9 \pm 0.3) \times 10^9$ |
| $CO_2^{\cdot-} + {}^+HAdH_2 \rightarrow CO_2 + {}^+HAdH_2^{\cdot-}$ | 6.5 | $(3.4 \pm 0.4) \times 10^9$ |
| $CO_2^{\cdot-} + AdH^- + H_2O \rightarrow CO_2 + AdH_2^{\cdot-} + OH^-$ | 11.5 | $(1.8 \pm 0.2) \times 10^8$ |
| $2\,{}^+HAdH_3^{\cdot} \rightarrow {}^+HAdH_2 +$ hydroquinone | 1.1 | $2k = (1.3 \pm 0.2) \times 10^9$ |
| $2AdH_2^{\cdot} \rightarrow AdH^-(Ad^{2-}) +$ hydroquinone | 13 | $2k = (5.6 \pm 0.8) \times 10^8$ |
| ${}^+HAdH_2^{\cdot-} + O_2 \rightarrow O_2^{\cdot-} + {}^+HAdH_2$ | 6.0 | $(3.5 \pm 0.4) \times 10^8$ |
| $AdH_2^{\cdot-} + O_2 + OH^- \rightarrow O_2^{\cdot-} + AdH^- + H_2O$ | 11.5 | $(1.7 \pm 0.2) \times 10^8$ |

**Figure 5** Oscilloscope/computer traces showing the formation of fully reduced adriamycin (hydroquinone) by mutual reaction of semiquinone free radicals. [adriamycin] = $4.9 \times 10^{-5}$ mol dm$^{-3}$ [formate] = $10^{-1}$ mol dm$^{-3}$, optical path length 2.5 cm, dose 7 Gy. (a) pH 5.0, $\lambda = 370$ nm; (b) pH 5.0; $\lambda = 400$ nm; (c) pH 5.0 $\lambda = 480$ nm; (d) pH 11.5, $\lambda = 430$ nm; (e) pH 11.5, $\lambda = 480$ nm; (f) pH 11.5, $\lambda = 520$ nm.

aqueous solution (Afanas'ev *et al.*, 1980). At biological pH values the reaction may be expressed:

$$^{+}HAdH_2^{\cdot -} + O_2 \rightarrow {}^{+}HAdH_2 + O_2^{\cdot -} \quad (15)$$

Equation (15) bears directly on a possible mode of action of adriamycin in which the drug is enzymically reduced (Arcamone, 1981; Bachur *et al.*, 1977; Crooke & Reich, 1980; Kalyanaraman *et al.*, 1980; El Khadem, 1982; Lown *et al.*, 1982*b*; Muggia *et al.*, 1982; Neidle & Waring, 1983) to the semiquinone and then regenerated by reaction with oxygen with concomitant formation of superoxide, $O_2^{\cdot -}$, from which hydrogen peroxide and hydroxyl radicals could arise and cause strand scission of DNA via abstraction of hydrogen from deoxyribose residues (Arcamone, 1981; Bates & Winterbourn, 1982; Crooke & Reich, 1980; El Khadem, 1982; Lown, 1979; Lown *et al.*, 1982*a,b*; Muggia *et al.*, 1982; Neidle & Waring, 1983; Winterbourn, 1981*b*).

Whilst the properties of the quinone and the semiquinone may be affected by intercalation, and therefore under these conditions not be precisely represented by the present work, evidence is accumulating (Tritton & Yee, 1982) that intercalation may not be essential for the anti-tumour action of adriamycin-like quinones. However, although the semiquinone itself has been implicated (Winterbourn, 1981*a*) the present results still point to a destructive mechanism based on superoxide formation, and in this respect may be particularly relevant to adriamycin cardiotoxicity (Green *et al.*, 1984; Lown *et al.*, 1982*a*).

### *Absorption spectrum of the hydroquinone*

Hydroquinone is present at relatively high concentration a few ms after delivery of pulses to solutions at pH values where reaction (14) is largely to the right. The absorption spectrum of the hydroquinone was determined at pH 5.0 and 11.5 using the known extinction coefficients of the parent ($\varepsilon_Q$) and the semiquinone ($\varepsilon_R$) assuming that the absorption at 720 nm is a measure of the semiquinone concentration, $a_e$. Typical traces are shown in Figure 5. At any wavelength, the observed increase in absorbance, $\Delta A$, is given by:

$$\Delta A = [a_e\varepsilon_R + \left(\frac{R_o - a_e}{2}\right)\varepsilon_{HQ} - ([Q_o] - [Q_e])\varepsilon_Q]d \quad (16)$$

where $R_o$ is the concentration of radicals initially produced, $[Q_o]$ and $[Q_e]$ are the initial concentration of quinone and the concentration of the quinone at the time of measurement, respectively, and $d$ is the optical path length. The absorption spectra obtained are shown in Figure 6. The principal uncertainty is in ascribing the absorption at 720 nm exclusively to the semiquinone. As the absorptions at 720 nm a few ms after the pulse were at their highest between pH ~6 and ~11 it was not possible to make a reliable determination of the absorption spectrum of the hydroquinone at pH values in this range. However the marked difference between the spectra at pH 5.0 and 11.5 shows that the hydroquinone exists in different protonated forms at these two pH values, so it has at least one $pK_a$ in this region in addition to that of the amino group, which would not be expected to appreciably influence the optical absorption.

**Figure 6** Absorption spectra of the hydroquinone obtained from traces like those in **Figure 5**, (○) pH 5.0, (●) pH 11.5.

### *Slow elimination of daunosamine from the hydroquinone*

Changes in absorption were observed for periods extending to some seconds after delivery of pulses (~8 Gy) to solutions containing $5 \times 10^{-5}$ mol dm$^{-3}$ adriamycin and $10^{-1}$ mol dm$^{-3}$ formate. At pH 9 the absorptions at 720 nm (largely semiquinone), 480 nm (parent and semiquinone) and 420 nm (mainly fully reduced form) decreased over hundreds of milliseconds, yielding species possessing strong absorptions at 380 and 608 nm. The data appeared to be consistent with loss of daunosamine from the hydroquinone, yielding a tautomer of 7-deoxyadriamycinone, analogous to the corresponding reaction seen after reduction of daunomycin in methanol by free radical electron donors (Kleyer & Koch, 1983). However there could be an intermediate step. Full spectroscopic and kinetic data were not obtained, but the results so far seem to be consistent with a rate constant of about $5 s^{-1}$ for the main process at pH 9. A somewhat similar conversion, occurring over a similar period, could be seen at pH 11.5, except that there was little semiquinone to disappear along with the hydroquinone. At pH 5, the rate of conversion was about a quarter of that seen in the more alkaline solutions. Further work will be required to establish the spectra of the tautomer and other possible intermediates as a function of pH, to establish a full kinetic scheme and to determine accurate rate constants.

This work was supported by grants from the Cancer Research Campaign and the Medical Research Council. The paper is dedicated to Professor Schulte-Frohlinde on the occasion of his 60th birthday.

### References

ADAMS, G.E., BOAG, J.W., CURRANT, J. & MICHAEL, B.D. (1965). The pulse radiolysis of aqueous solutions of thiocyanate ion. In *Pulse Radiolysis*, p. 117. (Eds. Ebert *et al.*) London: Academic Press.

AFANAS'EV, I.B., POLOZOVA, N.I. & SAMOKHVALOV, G.I. (1980). Investigation of the interaction of superoxide ion with adriamycin and the possible origin of cardiotoxicity of the anthracycline anticancer antibiotics. *Bioorg. Chem.*, **9**, 434.

ARCAMONE, F. (1981). *Doxorubicin Anticancer Antibiotics*. (Medicinal Chemistry Monographs, **17**) London: Academic Press.

BACHUR, N.R., GORDON, S.L. & GEE, M.V. (1977). Anthracycline antibiotic augmentation of microsomal electron transport and free radical formation. *Mol. Pharmacol.*, **13**, 901.

BACHUR, N.R., GORDON, S.L. & GEE, M.V. (1978). A general mechanism for microsomal activation of quinone anticancer agents to free radicals. *Cancer Res.*, **38**, 1745.

BACHUR, N.R., GORDON, S.L., GEE, M.V. & KON, H. (1979). NADPH cytochrome P-450 reductase activation of quinone anticancer agents to free radicals. *Proc. Natl Acad. Sci.*, **76**, 956.

BARTHELEMY-CLAVEY, V., MAURIZOT, J., DIMICOLI, J. & SICARD, P. (1974). Self-association of daunomycin, *FEBS Lett.*, **46**, 5.

BATES, D.A. & WINTERBOURN, C.C. (1982). Deoxyribose breakdown by the adriamycin semiquinone and $H_2O_2$: evidence for hydroxyl radical participation. *FEBS Lett.*, **145**, 137.

CROOKE, S.T. & REICH, S.D. (Eds.) (1980). *Anthracyclines. Current Status and New Developments*. New York: Academic Press.

EKSBORG, S. (1978). Extraction of daunorubicin and doxorubicin and their hydroxyl metabolites: self-association in aqueous solution. *J. Pharm. Sci.*, **67**, 782.

EL KHADEM, H.S. (Ed.). (1982). *Anthracycline Antibiotics*. New York: Academic Press.

GREEN, M.D., SPEYER, J.L. & MUGGIA, F. (1984). Cardiotoxicity of anthracyclines. *Eur. J. Cancer Clin. Oncol.*, **20**, 293.

HENRY, D.W. (1976). Adriamycin. In Cancer Chemotherapy. Symposium Series (Ed. A.C. Sartorelli) Washington, D.C.: *Am. Chem. Soc.*, **30**, 15.

HOFFMAN, D.M., GROSSANO, D.D., DAMIN, L.A. & WOODCOCK, T.M. (1979). Stability of refrigerated and frozen solutions of doxorubicin hydrochloride. *Am. J. Hosp. Pharm.*, **36**, 1536.

JOHNSTON, J.B., ZWELLING, L.A., KERRIGAN, D. LLOYD, L.S. & GLAZER, R.I. (1983). Comparison of DNA scission and cytotoxocity produced by adriamycin and 5-iminodoxorubicin in human colon carcinoma cells. *Biochem. Pharmacol.*, **32**, 2255.

KALYANARAMAN, B., PEREZ-REYES, E. & MASON, R.P. (1980). Spin trapping and direct electron spin resonance investigation of the redox metabolism of quinone anticancer drugs. *Biochim, Biophys. Acta*, **630**, 119.

KEENE, J.P. (1972). The linear accelerator at the Paterson Laboratories, Manchester. *Quad. dell'Area di Ricerca dell' Emilia-Romagna*, **1**, 49.

KLEYER, D.L. & KOCH, T.H. (1983). Spectroscopic observation of the tautomer of 7-deoxydaunomycinone from elimination of daunosamine from daunomycin hydroquinone. *J. Am. Chem. Soc.*, **105**, 2504.

LAND, E.J., MUKHERJEE, T., SWALLOW, A.J. & BRUCE, J.M. (1983*a*). One-electron reduction of adriamycin: properties of the semiquinone. *Arch. Biochem. Biophys.*, **225**, 116.

LAND, E.J., MUKHERJEE, T., SWALLOW, A.J. & BRUCE, J.M. (1983*b*). Reduction of the naphthazarin molecule as studied by pulse radiolysis. Part 1: addition of a single electron. *J. Chem. Soc. Faraday Trans. I*, **79**, 391.

LAND, E.J., MUKHERJEE, T. SWALLOW, A.J. & BRUCE, J.M. (1983*c*). Reduction of the napthazarin molecule as studied by pulse radiolysis. Part 2: second one-electron step. *J. Chem. Soc. Faraday Trans. I*, **79**, 405.

LOWN, J.W. (1979). The molecular mechanism of antitumour action of the mitomycins. In: Mitomycin *C. Current Status and New Developments*. p. 5. (Eds. Carter *et al.*) New York: Academic Press.

LOWN, J.W., CHEN, H.-H., PLAMBECK, J.A. & ACTON, E.M. (1982*a*). Further studies on the generation of reactive oxygen species from activated anthracyclines and the relationship to cytotoxic action and cardiotoxic effects. *Biochem. Pharmacol.*, **31**, 575.

LOWN, J.W., JOSHUA, A.V. & LEE, J.S. (1982*b*). Molecular mechanisms of binding and single-strand scission of deoxyribonucleic acid by the antitumor antibiotics saframycins A and C. *Biochemistry*, **21**, 419.

MUGGIA, F.M., YOUNG, C.W. & CARTER, S.K. (Eds.) (1982). *Anthracycline Antibiotics in Cancer Therapy* (Developments in Oncology, **10**) The Hague: Martinus Nijhoff.

NEIDLE, S. & WARING, M.J. (Ed.) (1983). *Molecular Aspects of Anti-Cancer Drug Action* (Topics in Molecular and Structural Biology, **3**). London: MacMillan.

ROSEN, G., WOLLNER, N., TAN, C. & 5 others (1974). Disease-free survival in children with Ewing's sarcoma treated with radiation therapy and adjuvant four-drug sequential chemotherapy. *Cancer*, **33**, 384.

SATO, S., IWAIZUMI, M., HANDA, K. & TAMURA, Y. (1977). Electron spin resonance study on the mode of generation of free radicals of daunomycin, adriamycin and carboqone in NAD(P)H-microsome system. *Gann*. **68**, 603.

SINHA, B.K. & GREGORY, J.L. (1981). Role of one-electron and two-electron reduction products of adriamycin and daunomycin in deoxyribonucleic acid binding. *Biochem. Pharmacol.*, **30**, 2626.

STURGEON, R.J. & SCHULMAN, S.G. (1977). Electronic absorption spectra and protolytic equilibria of doxorubicin: direct spectrophotometric determination of microconstants. *J. Pharm. Sci.*, **66**, 958.

SVINGEN, B.A. & POWIS, G. (1981). Pulse radiolysis studies of antitumor quinones: radical lifetimes, reactivity with oxygen, and one-electron reduction potentials. *Arch. Biochem. Biophys.*, **209**, 119.

SWALLOW, A.J. (1982). Physical chemistry of semiquinones. In *Function of Quinones in Energy Conserving Systems*, p. 59 (Ed. Trumpower) New York: Academic Press.

TRITTON, T.R. & YEE, G. (1982). The anticancer agent adriamycin can be actively cytotoxic without entering cells. *Science*, **217**, 248.

WINTERBOURN, C.C. (1981*a*). Cytochrome c reduction by semiquinone radicals can be indirectly inhibited by superoxide dismutase. *Arch. Biochem Biophys.*, **209**, 159.

WINTERBOURN, C.C. (1981*b*). Evidence for the production of hydroxyl radicals from the adriamycin semiquinone and $H_2O_2$. *FEBS Lett.*, **136**, 89.